# The effects of bariatric surgery on cardiac function: a systematic review and meta-analysis

**DOI:** 10.1038/s41366-023-01412-3

**Published:** 2023-11-25

**Authors:** Narek Sargsyan, Jun Yu Chen, Ravi Aggarwal, Michael G. Fadel, Matyas Fehervari, Hutan Ashrafian

**Affiliations:** 1 Department of General Surgery, Imperial College Healthcare NHS Foundation Trust, London, UK; 2 Department of Surgery and Cancer, Imperial College London, London, UK

**Keywords:** Cardiovascular diseases, Weight management, Obesity

## Abstract

**Introduction:**

Obesity is associated with alterations in cardiac structure and haemodynamics leading to cardiovascular mortality and morbidity. Culminating evidence suggests improvement of cardiac structure and function following bariatric surgery.

**Objective:**

To evaluate the effect of bariatric surgery on cardiac structure and function in patients before and after bariatric surgery.

**Methods:**

Systematic review and meta-analysis of studies reporting pre- and postoperative cardiac structure and function parameters on cardiac imaging in patients undergoing bariatric surgery.

**Results:**

Eighty studies of 3332 patients were included. Bariatric surgery is associated with a statistically significant improvement in cardiac geometry and function including a decrease of 12.2% (95% CI 0.096–0.149; *p* < 0.001) in left ventricular (LV) mass index, an increase of 0.155 (95% CI 0.106–0.205; *p* < 0.001) in E/A ratio, a decrease of 2.012 mm (95% CI 1.356–2.699; *p* < 0.001) in left atrial diameter, a decrease of 1.16 mm (95% CI 0.62–1.69; *p* < 0.001) in LV diastolic dimension, and an increase of 1.636% (95% CI 0.706–2.566; *p* < 0.001) in LV ejection fraction after surgery.

**Conclusion:**

Bariatric surgery led to reverse remodelling and improvement in cardiac geometry and function driven by metabolic and haemodynamic factors.

## Introduction

Obesity is a global health issue with a plethora of medical and socioeconomic implications that continues to increase in prevalence. It is predicted that one in five women and one in seven men will be living with obesity by 2030 [1]. Obesity leads to the development of cardiovascular risk factors and cardiovascular disease mortality [2]. The adverse effects of obesity on the cardiovascular system can lead to intrinsic cardiac changes including an increase in left ventricular mass (LVM), left ventricular hypertrophy, and ventricular and atrial dilatations. These obesity-related structural changes combined with metabolic syndrome can cause impaired cardiac function as a result of maladaptive cardiac remodelling [3–6].

Current literature suggests that weight loss may lead to reverse cardiac remodelling and improve cardiac structure and function [7]. Bariatric surgery has been recognised as the most effective and sustainable long-term intervention to achieve weight loss in patients with severe obesity. In addition to the reduction in metabolic syndrome, the beneficial effects of bariatric surgery on cardiac structure and function have been demonstrated in various studies [8–12]. The aim of this systematic review and meta-analysis is to quantify the impact of bariatric surgery on changes to cardiac structure and function by synthesising extensive data available in the literature.

## Methods

This systematic review was performed in line with a registered protocol and reported according to Preferred Reporting Items for Systematic Reviews and Meta-Analyses (PRISMA) [13]. The review was also registered on PROSPERO Centre for Reviews and Dissemination (registration number CRD42023396430).

A literature search was performed in August 2022 using MEDLINE (via PubMed), EMBASE (via OVID) and Cochrane database using MeSH terms in all combinations: ‘bariatric surgery’ or ‘metabolic surgery’ or ‘weight loss’ or ‘obesity surgery’ and ‘echocardiography’ or ‘magnetic resonance imaging’ or ‘cardiac imaging’ or ‘cardiac dimensions’ or ‘ventricular dimensions’ or ‘cardiac function’ or ‘cardiac structure’. Studies identified from the search strategy were entered into Covidence (Victoria, Australia) for duplication removal and bibliographic management. Authors NS, LA and JYC independently identified relevant studies and discrepancies were resolved by consensus between all authors. The exact search strategy is outlined in Supplementary material 1.

### Inclusion and exclusion criteria

The inclusion and exclusion criteria were defined before commencement of the literature search. The following criteria were required for inclusion in the study:

i. Randomised controlled trials (RCT), prospective or retrospective cohort studies, case (control) studies, cross-sectional studies.
ii. Patients who had undergone bariatric surgery and had an echocardiogram pre- and postoperatively.
iii. Reported outcomes of interest including cardiac geometry, systolic and diastolic function.
iv. Original, full publications published in the English language.

All studies reporting echocardiographic or magnetic resonance cardiac structural or functional parameters were included. Studies were excluded from analysis if extraction of data was not possible due to inconsistencies, defined as the lack of reported pre- and/or postoperative data.

### Data extraction and quality assessment

A standardised data extraction form was developed on Covidence and two authors (NS and JYC) independently extracted all relevant data. All discrepancy was resolved by group discussion. Quality scoring of studies was performed using the Newcastle–Ottawa assessment tool.

### Statistical analysis

Data analysis was performed using Stata Software, Version 15.1. StataCorp LCC, TX. Random effects analysis was used to calculate weighted mean difference and mass effect. All studies were included in the analysis if relevant data were available. Data were analysed using a random effects model and statistical heterogeneity was calculated using *I*^2^. An *I*^2^ of <30 was considered as low, 30–60 as moderate and >60 as high heterogeneity. Results were computed and represented on forest plots (see supplemented section).

## Results

Eighty studies were found to fulfil the inclusion criteria and were included in this systematic review, producing a pooled patient population of 3332 patients. Fifty-four were prospective studies and 26 were retrospective studies. The average quality of studies according to the Newcastle–Ottawa scale was good (6.4). The mean age was 42 years old with a mean starting BMI of 45.9 kg/m^2^. The mean follow-up period was 12.2 [1–58] months. The study characteristics are summarised in Table 1.

**Table 1:**
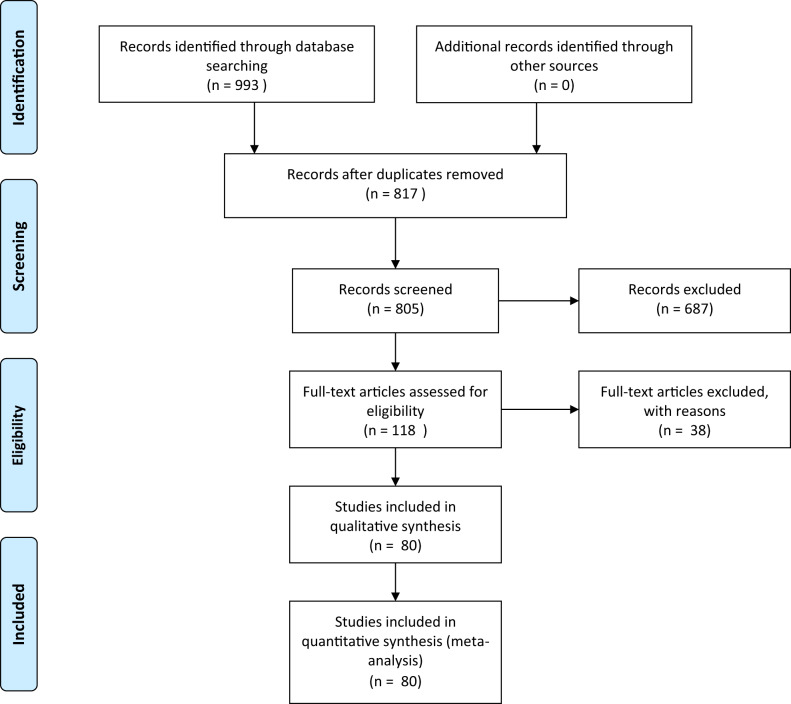
Outline of search strategy according to the PRISMA guidelines.

### Cardiac geometry

#### Left ventricular mass (LVM)

**Fig. 1:**
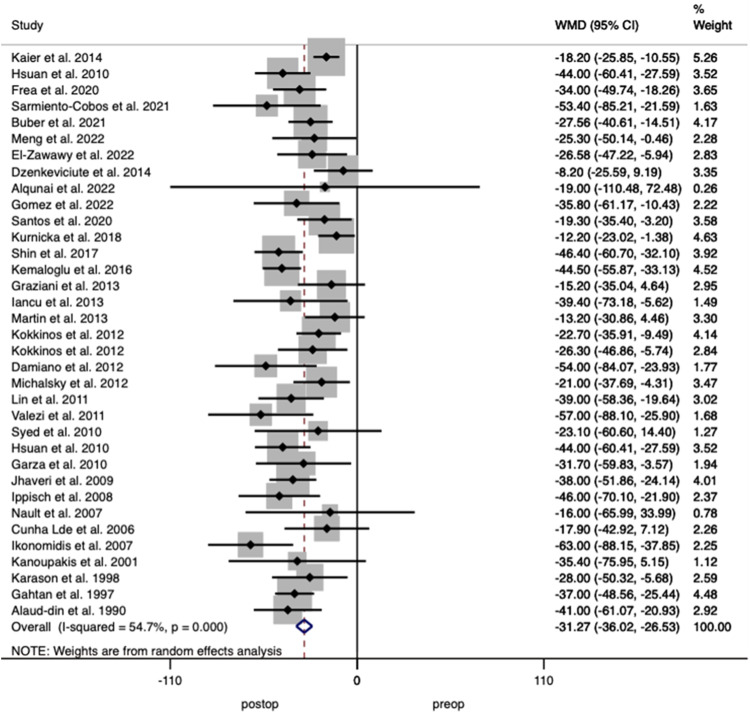
Forest plot demonstrating changes in left ventricular mass before and after bariatric surgery.

Absolute LVM was reported in 35 studies, which included 2369 patients. Pooled analysis demonstrated a mean decrease of 31.27 g in LVM after surgery (95% CI 26.53–36.12; *p* < 0.001). There was a moderate heterogeneity between studies of *I*^2^ = 54.7% (Fig. 1).

#### Left ventricular mass index (LVMI)

**Fig. 2:**
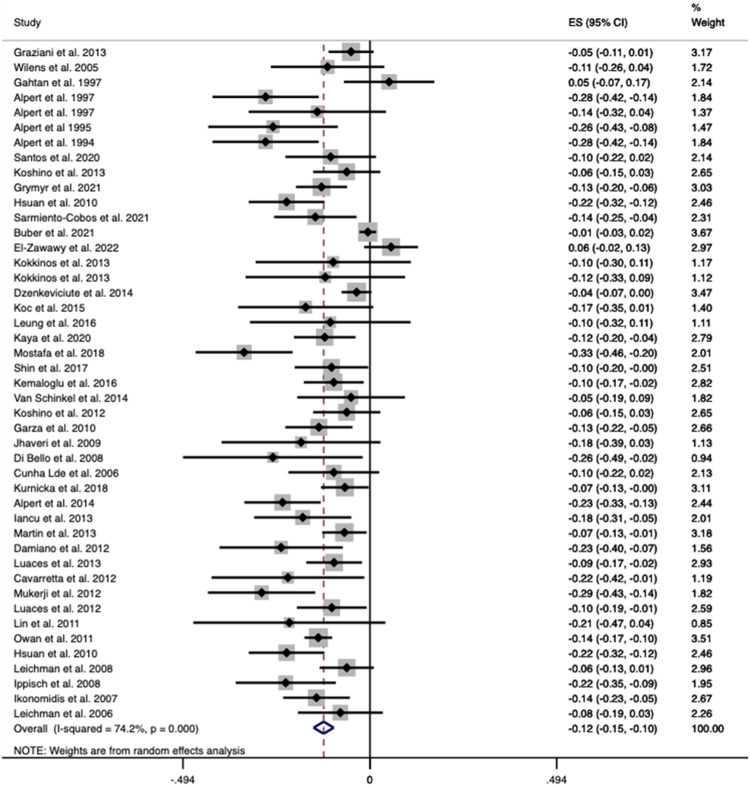
Forest plot demonstrating changes in left ventricular mass index before and after bariatric surgery.

LVMI is calculated to standardised measurements of LVM. It is often recommended that height is raised to the power of 2.7 (m^2.7^) as described by deSimone et al. [59]. This method accounts for close to the equivalent of the effect of lean body mass and excludes the effect of obesity and blood pressure elevation on LVM [60]. Twenty-two studies including 1142 patients reported on LVMI relative to body surface area (m^2^), analysis showed a decrease after surgery of 10.13% (95% CI 4.270–15.996; *p* < 0.001); 7 studies including 198 patients, indexed to height (m) showed a decrease of 19.26% (95% CI 1.815–40.336; *p* < 0.001) and 16 studies including 1021 patients indexed to height (m^2.7^) showed a decrease of 8.66% (95% CI 6.347–10.977; *p* < 0.001). Proportional analysis of findings in 45 studies with 2361 patients demonstrated a weighted mean decrease of 12.2% (95% CI 0.096–0.149; *p* < 0.001) in LVMI after surgery with heterogeneity of *I*^2^ = 74.2% (Fig. 2).

#### Left ventricular end-systolic volume (LVESV)

**Fig. 3:**
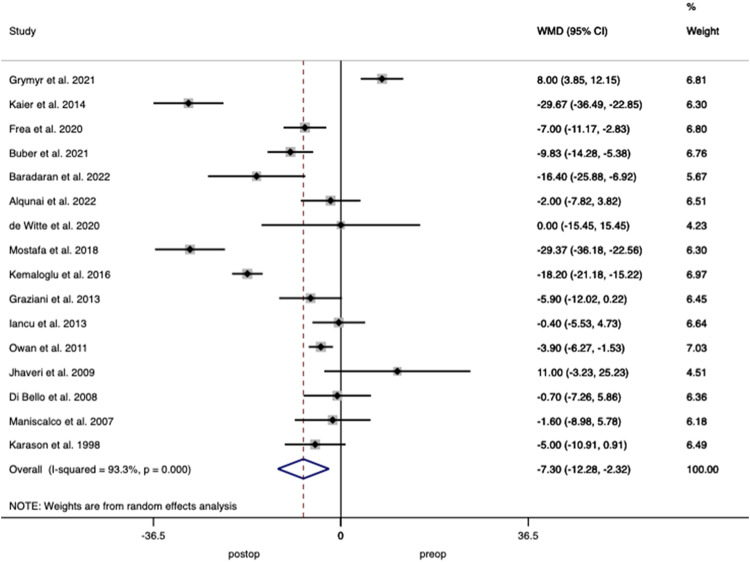
Forest plot demonstrating changes in left ventricular end-systolic volume before and after bariatric surgery.

Sixteen studies including 1024 patients reported on LVESV demonstrating a weighted mean decrease of 7.29 mL (95% CI 2.31–12.27; *p* = 0.004) after surgery, with a heterogeneity score of *I*^2^ = 93.3% (Fig. 3).

#### Left ventricular end-diastolic volume (LVEDV)

**Fig. 4:**
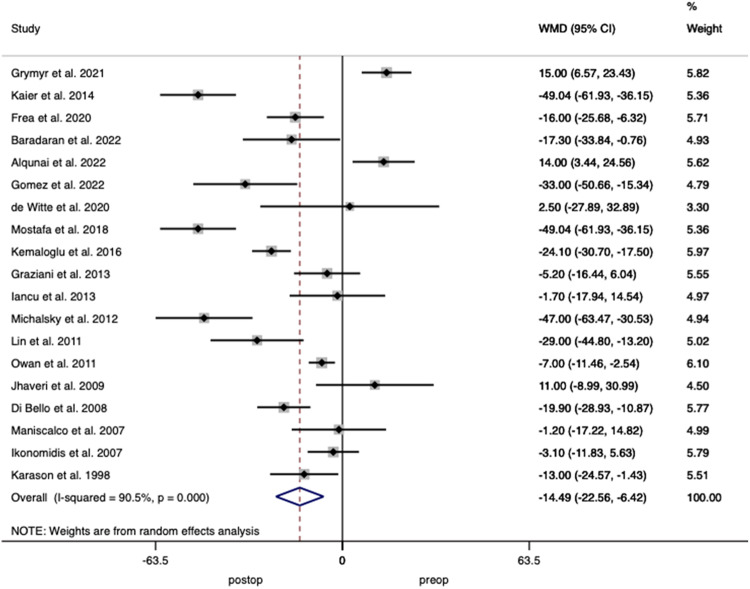
Forest plot demonstrating changes in left ventricular end-diastolic volume before and after bariatric surgery.

Nineteen studies including 1111 patients reported on LVEDV demonstrating a weighted mean decrease of 14.49 mL (95% CI 6.42–22.56; *p* < 0.001) after surgery with heterogeneity of *I*^2^ = 90.5% between studies (Fig. 4).

#### Left ventricular global longitudinal strain (LVGLS)

**Fig. 5:**
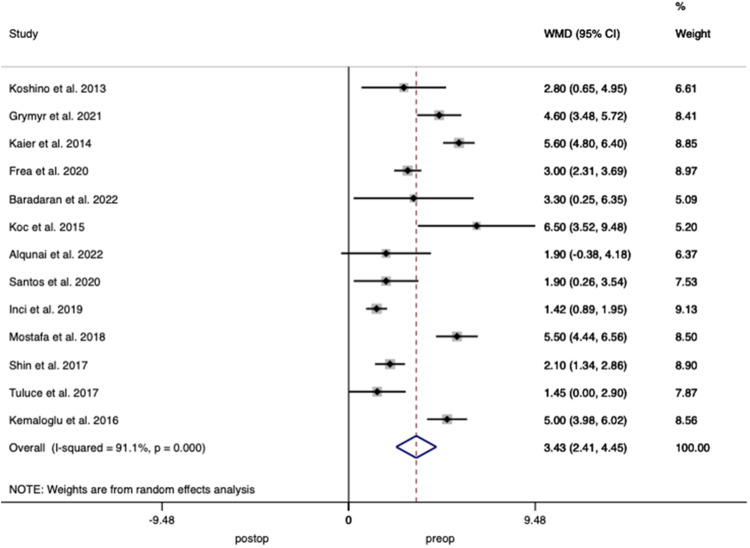
Forest plot demonstrating changes in left ventricular global longitudinal strain before and after bariatric surgery.

Thirteen studies including 539 patients reported LVGLS demonstrating a weighted mean increase of 3.43% (95% CI 2.41–4.45; *p* < 0.001), with a heterogeneity of *I*^2^ = 91.1% between studies (Fig. 5).

#### Left ventricular systolic dimension (LVSD)

Twenty-four studies including 993 patients reported on LVSD pooled analysis demonstrated a decrease of 0.51 mm (95% CI 0.08–1.11; *p* < 0.001) after surgery, with a heterogeneity of *I*^2^ = 74.4% between studies.

#### Left ventricular diastolic dimension (LVDD)

**Fig. 6:**
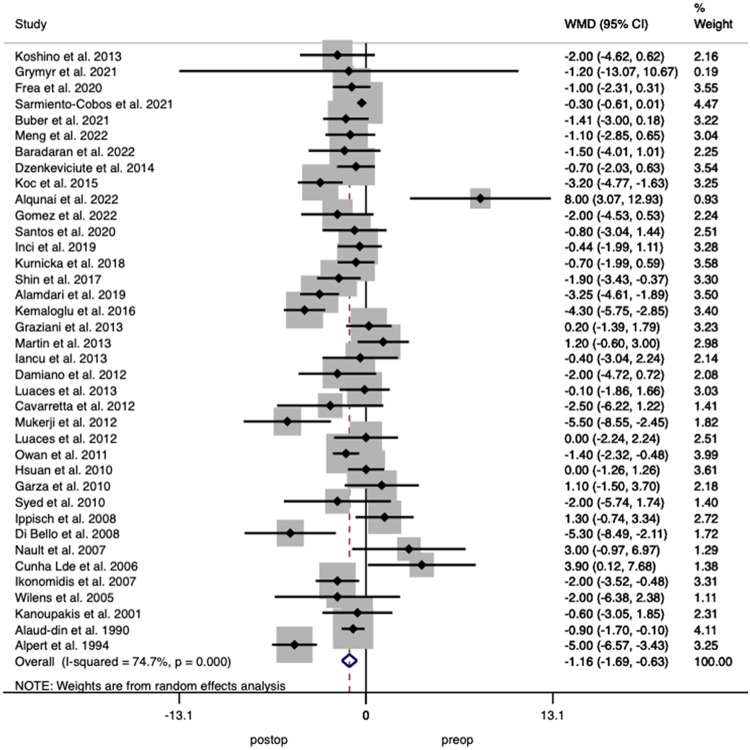
Forest plot demonstrating changes in left ventricular diastolic dimension before and after bariatric surgery.

Thirty-eight studies including 2071 patients reported on LVDD with analysis demonstrating a decrease of 1.16 mm (95% CI 0.62–1.69; *p* < 0.001) after surgery with a heterogeneity between studies of *I*^2^ = 74.7% (Fig. 6)

### Diastolic function

#### E/A ratio

The E/A ratio represents the ratio of peak velocity blood flow from left ventricular relaxation in early diastole (the E wave) to peak velocity flow in late diastole (the A wave) caused by atrial contraction [61]. It is therefore a marker of the function of the left ventricle of the heart. Thirty-six studies including 680 patients reported on the E/A ratio before and after surgery, demonstrating an increase of 0.155 (95% CI 0.106–0.205; *p* < 0.001) with interstudy heterogeneity of *I*^2^ = 77.1%.

#### Left atrial diameter (LA diameter)

Twenty-five studies including 1125 patients reported on LA diameter with pooled analysis demonstrating a decrease of 2.012 mm (95% CI 1.356–2.669; *p* < 0.001) after surgery with a heterogeneity between studies of *I*^2^ = 82.1%.

#### Septal E/A ratio

Five studies including 209 patients reported on septal E/A ratio with pooled analysis demonstrating a decrease of 0.797 (95% CI 0.234–1.361; *p* < 0.001) after surgery.

### Systolic function

#### Left ventricular ejection fraction (LVEF)

**Fig. 7:**
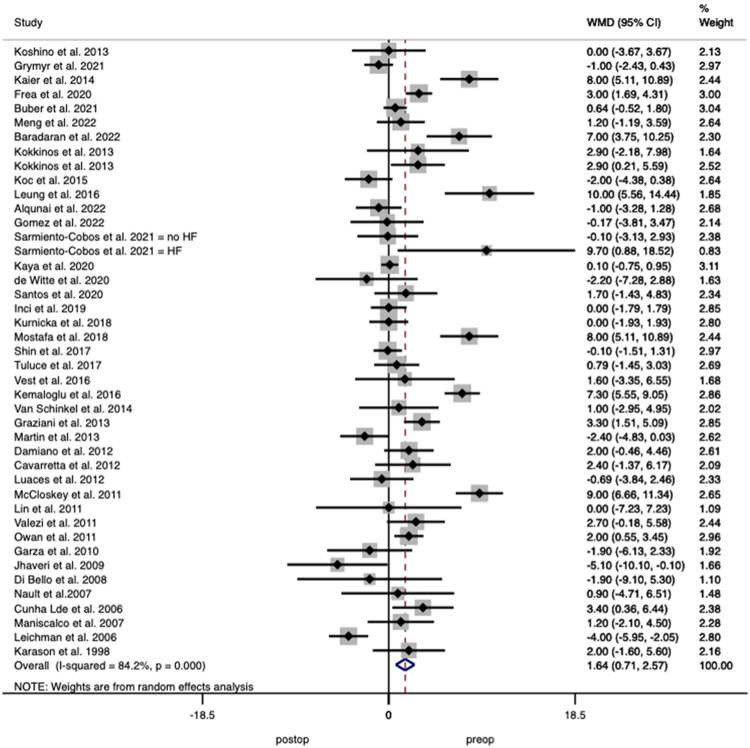
Forest plot demonstrating changes in left ventricular ejection fraction before and after bariatric surgery.

Forty-three studies including 1955 patients reported on LVEF with pooled analysis demonstrating an increase of 1.636% (95% CI 0.706–2.566 *p* < 0.001) after surgery, with interstudy heterogeneity of *I*^2^ = 84.2% (Fig. 7)

#### Stroke volume (SV)

Eight studies including 676 patients reported on SV demonstrating a decrease of 2.95 mL (95% CI –8.565 to 2.660; *p* < 0.001) after surgery with an interstudy heterogeneity of *I*^2^ = 72.6.

#### Cardiac output (CO)

Three studies including 151 patients reported on cardiac output demonstrating a decrease of 0.625 mL/min (95% CI –1.261 to 0.010; *p* = 0.089) after surgery with heterogeneity between studies of *I*^2^ = 58.7%.

#### Blood pressure

Resting systolic blood pressure was reported in 24 studies including 1066 patients. Pooled analysis demonstrated a decrease of 10.918 mmHg (95% CI 8.457–13.379; *p* < 0.001) after surgery with heterogeneity of *I*^2^ = 77.8% between studies. Resting diastolic blood pressure was reported in 23 studies including 1031 patients. Pooled analysis demonstrated a decrease of 5.921 mmHg (95% CI 4.646–7.795; *p* < 0.001) after surgery with heterogeneity of *I*^2^ = 75.8% between studies.

#### Heart rate

Resting heart rate was reported in 12 studies including 554 patients. Pooled analysis demonstrated a weighted mean decrease of 6.68 beats/min (95% CI 3.76–9.59; *p* < 0.001) after surgery with heterogeneity between studies of *I*^2^ = 86.1%.

## Discussion

This study demonstrates an improvement in cardiac structures and function in patients after undergoing bariatric surgery. These statistically significant improvements are reported in cardiac geometry, systolic and diastolic function across cardiac imaging modalities. For example, this was reflected by bariatric surgery leading to an increase in LVEF and E/A ratio, and a decrease in LA diameter, LVDD and LVMI. This systematic review and meta-analysis including eighty studies represents findings confirming and extending previous evidence in improvements in cardiac geometry and function, demonstrating statistically significant changes across all parameters.

### Cardiac geometry

Obesity has long been recognised as an independent risk factor to directly alter cardiac structure and function, including ventricular and septal hypertrophy leading to increased LVM and MACE (major adverse cardiovascular event) [5, 14]. Our results demonstrate a statistically significant weighted mean decrease of 12.2% in LVMI after bariatric surgery. There was also a mean weighted decrease of 31.27 g in LV mass as a measure of improvement of left ventricular hypertrophy (LVH). This supports the notion that bariatric surgery leads to LV reverse remodelling and plasticity, resulting in a long-term decrease in cardiovascular mortality in bariatric patients [3, 15].

The metabolic effects of bariatric surgery is thought to improve the haemodynamic parameters and lead to reverse remodelling by decreasing the inflammatory state that induces ventricular hypertrophy associated with pathological diastolic dysfunction and ventricular filling, reduced ventricular contractility, reduced coronary reserve, chronic overload and arrhythmogenic electrical dysfunction [16, 17].

The concept of beneficial reverse remodelling of LVH following bariatric surgery is further supported by our results, demonstrating an improvement in posterior wall thickness, interventricular septal thickness and relative wall thickness. In addition, our study demonstrated improvement in end-systolic and end-diastolic volume and diameter dimensions supporting increased filling and relaxation of the left ventricles after bariatric surgery.

### Diastolic function

Our study demonstrates that bariatric surgery can lead to improvement in echocardiographic markers of diastolic dysfunction through reverse remodelling of obesity-related changes in cardiac structure and function influenced by neurohormonal and metabolic factors. Diastolic dysfunction with impaired myocardial relaxation is characterised by a decreased early (E wave) but enhanced atrial LV filling (A wave). Thus, atrial contraction basically compensates for the decrease in early passive filling and results in lower E/A ratio. Our study shows a statistically significant increase in E/A ratio post bariatric surgery; significant decrease in LA diameter, which is an important measure of diastolic function as LA enlargement reflects chronic exposure to LV filling pressure. In addition, LVMI is also a predictor of LA enlargement. An increased LA diameter is associated with an increased risk of developing atrial fibrillation so an improvement in these parameters after bariatric surgery reflects a positive impact on diastolic function and arrhythmogenesis. LA diameter is also known to be associated with obstructive sleep apnoea, which has also been demonstrated to be reported to improve in patients after undergoing bariatric surgery [18, 19].

### Systolic function

The development of LV systolic dysfunction has long been recognised as a complication from severe obesity. Our meta-analysis demonstrated a significant increase in LVEF after bariatric surgery. While bariatric surgery has been shown to improve diastolic dysfunction, the effect on LV systolic function and mechanics has been little explored. Previous studies demonstrated that improvement in systolic function only occurs in obese patients with systolic dysfunction preoperatively and those individuals who have been severely obese for a longer period of time [20, 21]. More recently, rapid weight loss from bariatric surgery has been shown to be associated with a considerable increase in LVEF and significant improvement in systolic function, especially in patients with pre-existing heart failure [22] and even in those individuals indicated for a cardiac transplant [23].

Our systematic review and meta-analysis has also demonstrated an improvement in LVGLS post bariatric surgery, which is used as a marker of left systolic mechanics. Postoperative increase in GLS is associated with postoperative reduction in afterload, mean blood pressure and BMI [24]. Improvement in parameters of LV geometry and mechanics in our study demonstrated an improvement in systolic dysfunction in patients after bariatric surgery. These profound improvements in cardiac imaging after bariatric surgery can be related to the mechanisms in how these procedures achieve beneficial reverse remodelling.

Adipose tissue has been considered as an endocrine organ with the ability to produce a variety of hormones and cytokines [25, 26]. Therefore, the combined effects of weight loss and metabolic enhancement from bariatric surgery can result in reverse remodelling of cardiac geometry and function. Obesity is also linked with sympathetic nervous system overactivity [27] and baroreflex sensitivity [28], and this cardiac autonomic nervous system imbalance results in increased heart rate and hypertension. Our meta-analysis has demonstrated a significant reduction in heart rate and blood pressure.

The metabolic and haemodynamic hypothesis as described by le Roux et al. [29] may explain the beneficial surgical effects in morbid obesity; metabolites such as leptin and other adipokines are reported to induce ventricular hypertrophy in obesity and lead to increasing circulating blood volume contributing to ventricular dilatation and hypertrophy [30]. Bariatric surgery leads to reversal of metabolic dysfunction which in turn can contribute to improved cardiac structure and function.

Bariatric surgery causes several metabolic improvements described as BRAVE effect: bile flow alterations, reduction of gastric size, anatomical gut rearrangement and altered flow of nutrients, vagal manipulation, enteric gut modulation [31], which contributes to cardiac reverse remodelling. These effects occur almost simultaneously after surgery and may offer a paradigm to identify the profound mechanisms driving improvements in glucose metabolism, insulin resistance, gut hormone release, microbiota and adipokine modulation [32–35]; which may offer a resolution of obesity-linked cardiac dysfunction.

The impact of bariatric surgery on enteric gut hormones has been reported to have a beneficial effect on cardiac function via the entero-cardiac axis [36, 37]. By inducing changes in secretion of hormones such as secretin produced in the duodenum, glucagon produced in the pancreas and vasoactive intestinal peptide produced in pancreas, gut and brain, act as inotropes indirectly involved in cardiac cell communication by activating cardiac membrane adenylate cyclase. The exact mechanism is currently unclear, although it is thought that cardiac energy metabolism is enhanced through tricarboxylic acid (TCA) cycle intermediaries, cardiorenal protective activities and biochemical caloric restrictions [29, 38].

Anorexigenic gut hormones, mainly GLP-1 (glucagon-like peptide-1) and peptide YY associated with improvement in insulin secretion and sensitivity, have been found to favourably modulate cardiac function by reducing vascular tone and increase in myocardial contractility. More recently, cardiovascular outcome trials (CVOTs) of GLP-1 receptor agonists have demonstrated potential cardiovascular protective potential by retarding lipotoxicity and cardiac inflammation via pyroptotic cytokines but also improving cardiac energy metabolism [29, 37–40].

The orexigenic hormone ghrelin has been suggested to improve SV index, ejection fraction and cardiac index with a decrease in LV wall stress. Adipokines such as leptin, TNFα and adiponectin have demonstrated cardiovascular activity with circulating leptin levels correlating with LV mass in morbid obesity and may also be responsible in contributing to the beneficial cardiac effects of bariatric surgery [37, 41–43]. The renin-angiotensin-aldosterone system (RAAS) can counter haemodynamic stress. However, sustained activation of RAAS and angiotensin release worsens cardiac fibrosis, cellular necrosis and induces cardiomyocyte hypertrophy, inhibition of RAAS is linked with reverse cardiac remodelling. Bariatric surgery has been linked to changes in several RAAS components, including a significant decrease in plasma renin activity, aldosterone and angiotensin-converting enzyme-2 (ACE2) activity and increase in ACE/ACE2 ratio [44–46].

Beta-adrenergic receptor blockade has demonstrated reverse LV remodelling and improvement in EF linked to SERCA2a, which has been linked to reversing cardiac excitation-contraction-coupling abnormalities and associated chamber dilatation and dysfunction [14, 22, 47, 48, 62–111]. Osteopontin (OPN) has emerged as a key ubiquitous phosphoprotein involved in numerous biological functions, including biomineralisation, extracellular matrix and tissue remodelling and repair. Enhanced expression of OPN appears to play an important role in remodelling of the heart following myocardial infarction as well as LVH. Bariatric surgery has been shown to increase OPN plasma concentration [112].

### Strengths and limitations

The strength of this study includes comprehensive data collection involving extensive data published in the literature and rigorous data extraction and statistical analysis. There are important limitations which must be addressed including the vulnerability of echocardiographic studies to subjectivity and reporting biases on interpretation by healthcare professionals undertaking and/or reporting cardiac imaging. In addition, the majority of the studies included were prospective studies, rather than RCTs, with a limited follow-up period.

## Conclusions

This meta-analysis and systematic review on the effect of bariatric surgery on cardiac structure and function assessed on cardiac imaging suggests that bariatric surgery is associated with significant improvement in cardiac structure and function. Bariatric surgery can drive reverse cardiac remodelling improving cardiac dysfunction. Further RCTs are justified to describe the relationship between the improvement of cardiac structure and function as assessed by imaging modalities to mortality and morbidity when addressing obesity-associated cardiac disease.

### Supplementary information

Supplementary Material Table 1

Data Set 1
