# Supplementary material for: The effects of bariatric surgery on cardiac function: a systematic review and meta-analysis
Source: Int J Obes (Lond). 2023 Nov 25;48(2):166–76. doi: 10.1038/s41366-023-01412-3 (PMC10824663; doi:10.1038/s41366-023-01412-3)
Supplement: Supplementary file 1 — Supplementary Material Table 1 [file 41366_2023_1412_MOESM1_ESM.docx]

*Supplementary Material*

Sup 1. Bariatric surgical studies and characteristics reporting on changes in cardiac structure and function

| **First author and year** | **Study design** | **Sample size** | **Age (mean)** | **Follow-up period (months)** | **Pre-op Mean starting BMI** | **Post-op mean BMI** | **Study Quality Score** |
| --- | --- | --- | --- | --- | --- | --- | --- |
| Koshino et al. 2013 [48] | Prospective | 29 | 52 | 23 | 51 | 37 | 7 |
| Grymyr et al. 2021 [24] | Prospective | 106 | 42 | 60 | 41.9 |  | 8 |
| Grymyr et al. 2021 [24] | Prospective | 94 | 43 | 14 | 41.8 | 28.8 | 8 |
| Kaier et al. 2014 [49] | Prospective | 52 | 44 | 6 | 42.4 | 31.5 | 8 |
| Hamo C et al. 2020 [50] | Retrospective | 98 | 45 | 12 | 47.1 |  | 6 |
| Hsuan et al. 2010 [14] | Prospective | 66 | 31 | 4 | 43.3 | 34.1 | 7 |
| Frea et al. 2020 [51] | Prospective | 40 | 42 | 10 | 44 | 31 | 8 |
| Sarmiento-Cobos et al. 2021 [22] | Retrospective | 40 | 64 | 12 | 39.9 | 33.1 | 8 |
| Buber et al. 2021 [52] | Prospective | 44 | 36 | 7 | 44.03 | 30.43 | 8 |
| Meng et al. 2022 [53] | Prospective | 44 | 32 | 12.28 | 41.6 | 30.3 | 8 |
| El-Zawawy et al. 2022 [54] | Prospective | 40 | 33 | 12 |  |  | 8 |
| Baradaran et al. 2022 [55] | Cross-sectional | 22 |  | 9 | 45 | 33.3 | 6 |
| Kardassis et al. 2012 [56] | Retrospective | 44 | 60 | 10 | 40 | 31.5 | 6 |
| Kokkinos et al. 2013 (RYGB) [57] | Prospective | 14 | 38 | 6 | 47.9 | 34.5 | 7 |
| Kokkinos et al. 2013 (GB) [57] | Prospective | 23 | 40 | 6 | 51.6 | 38.3 | 7 |
| Kardassis et al. 2013 [6] | Retrospective case-control | 19 | 50 | 10 | 40.6 | 31.2 | 6 |
| Dzenkeviciute et al. 2014 [58] | Prospective | 83 | 46 | 12 | 46.9 | 40.1 | 7 |
| Koc et al. 2015 [59] | Prospective | 17 | 36 | 6 | 44 | 38 | 8 |
| Leung et al. 2016 [60] | Prospective | 8 | 56 | 9 | 44 | 35 | 8 |
| Vest et al. 2016 [23] | Retrospective | 42 | 52 | 12 | 48.2 | 35.59 | 8 |
| Kemaloglu et al. 2016 [61] | Prospective | 53 | 37 | 6 | 49.1 | 36.9 | 8 |
| Brownell et al. 2016 [62] | Retrospective | 312 | 39 | 3 |  |  | 6 |
| Malekpour Alamdari et al. 2019 [63] | Prospective | 101 | 37 | 12 | 45.15 |  | 7 |
| Tuluce et al. 2017 [64] | Prospective | 32 | 34 | 1 | 43.95 | 38.92 | 6 |
| Shin et al. 2017 [65] | Prospective | 37 | 36 | 1 | 39.7 | 27.9 | 8 |
| Kurnicka et al. 2018 [66] | Prospective | 60 | 37 | 6 | 47 | 34 | 8 |
| Mostafa et al. 2018 [67] | Prospective | 52 | 38 | 6 | 42.3 | 28.5 | 7 |
| Inci et al. 2019 [68] | Prospective | 37 | 34.9 | 6 | 44.08 |  | 6 |
| Santos et al. 2020 [69] | Prospective | 25 | 35 | 4 | 46.8 | 38.4 | 7 |
| de Witte et al. 2020 [70] | Prospective | 15 | 48 | 12 | 40.1 | 27.5 | 8 |
| Kaya et al. 2020 [71] | Prospective | 61 | 37 | 6 | 47.1 | 37.1 | 8 |
| Sarmiento-Cobos et al. 2021 (no HF) [72] | Retrospective | 49 | 63 | 18 | 40.9 | 33.3 | 8 |
| Sarmiento-Cobos et al. 2021 (HF) [72] | Retrospective | 19 | 57 | 18 | 41.3 | 30.8 | 8 |
| Sarmiento-Cobos et al. 2022 [73] | Retrospective | 81 | 55 | 12 | 42.97 | 30.9 | 8 |
| Gomez et al. 2022 [15] | Retrospective | 51 | 63 | 16 | 40.3 | 31.8 | 6 |
| Alqunai et al. 2022 [74] | Prospective | 59 | 37 | 6 | 45.8 | 32.7 | 8 |
| Mukerji et al. 2012 [75] | Prospective | 39 | 37 | Nadir of weight loss | 42.8 | 31.9 | 8 |
| Alpert et al. 2015 [76] | **Prospective** | **67** | **38** | **5** | **46.2** | **34.5** | 6 |
| Kaier et al. 2014 [77] | Prospective | 52 | 44 | 6 | 42.4 | 31.5 | 6 |
| Van et al. 2014 [78] | Prospective | 9 | 54 | 4 | 41.3 | 34.1 | 6 |
| Graziani et al. 2013 [79] | Prospective | 51 | 63 | 24 | 47.9 | 35.7 | 7 |
| Iancu et al. 2014 [80] | Prospective | 34 | 39 | 12 | 43.6 | 28.9 | 5 |
| Martin et al. 2013 [81] | Prospective | 70 | 42 | 12 | 49.4 | 30.9 | 7 |
| Kokkinos et al. 2013 [82] | Prospective | 14 | 38 | 6 | 47.9 | 34.5 | 5 |
| Kokkinos et al. 2013 [82] | Prospective | 23 | 40 | 6 | 51.6 | 38.3 | 5 |
| Damiano et al. 2012 [83] | Prospective | 26 | 41 | 8 | 49.7 | 39.9 | 5 |
| Luaces et al. 2012 [84] | Prospective | 61 | 41 | 12 | 47.4 | 30.6 | 4 |
| Koshino et al. 2012 [85] | Retrospective | 28 | 52 | 22.7 | 51 | 37 | 7 |
| Cavarretta et al. 2013 [86] | Retrospective | 16 | 46 | 16 | 44.8 | 31.2 | 5 |
| Mukerji et al. 2012 [87] | Retrospective | 39 | 37 | 24 | 42.8 | 31.9 | 5 |
| Luaces et al. 2012 [88] | Prospective | 41 | 40 | 12 | 47.41 | 30.43 | 6 |
| Michalsky et al. 2013 [89] | Retrospective | 10 | 17 | 9.4 | 50.33 | 34.6 | 6 |
| Kardassis et al. 2012 [90] | Prospective | 44 | 60 | 12 | 40 | 31.5 | 6 |
| McCloskey et al. 2007 [91] | Retrospective | 14 | 46 | 6 | 50.8 | 36.8 | 4 |
| Lin et al. 2011 [92] | Prospective | 10 | 44 | 16 | 44 | 29 | 5 |
| Valezi et al. 2011 [93] | Prospective | 43 | 36 | 12 | 41.8 | 28.4 | 5 |
| Owan et al. 2011 [94] | Prospective | 338 | 42 | 24 | 47.9 | 32.2 | 8 |
| Algahim et al. 2010 [95] | Prospective | 15 | 49 | 24 | 46.7 | 32.4 | 6 |
| Syed et al. 2010 [96] | Prospective | 22 |  | 6 | 44 | 34.7 | 5 |
| Hsuan et al. 2010 [97] | Prospective | 66 | 31 | 3 | 43.3 | 34.1 | 5 |
| Garza et al. 2010 [98] | Retrospective | 57 | 51 | 45 | 49 | 35 | 7 |
| Jhaveri et al. 2009 [99] | Prospective | 13 | 45 | 17 | 44.1 | 29.9 | 5 |
| Leichman et al. 2008 [100] | Prospective | 43 | 45 | 3 | 51 | 43.3 | 5 |
| Ippisch et al. 2008 [101] | Retrospective | 38 | 16 | 10 | 60 | 40 | 6 |
| Di Bello et al. 2008 [102] | Prospective | 13 | 31 | 24 | 47 | 36 | 4 |
| Nault et al. 2007 [103] | Prospective | 10 | 38 | 6.8 | 52.3 | 37.7 | 6 |
| Maniscalco et al. 2007 [104] | Prospective | 12 | 43.5 | 12 | 43.2 | 31.7 | 6 |
| Cunha et al. 2006 [105] | Prospective | 23 | 38 | 36 | 48.8 | 31.8 | 5 |
| Ikonomidis et al. 2007 [106] | Prospective | 60 | 35 | 36 | 48.68 | 32 | 8 |
| Leichman et al. 2006 [107] | Prospective | 22 | 44 | 3 | 46.8 | 40.1 | 4 |
| Wilens et al. 2005 [108] | Retrospective | 17 | 43 | 7.4 | 54 | 40 | 5 |
| Kanoupakis et al. 2001 [109] | Prospective | 16 | 32.5 | 6 | 49 | 34 | 5 |
| Karason et al. 1998 [110] | Prospective | 38 | 48 | 12 | 39 | 29 | 7 |
| Gahtan et al. 1997 {111] | Prospective | 13 | 39 | 18 | 52.5 | 35.7 | 5 |
| Alpert et al. 1997 [112] | Retrospective | 39 | 37 | 4.5 | 50.4 | 30.9 | 3 |
| Alpert et al. 1997 [113] | Retrospective | 14 | 38 | 4.5 | 47 | 31.6 | 3 |
| Alpert et al. 1995 [21] | Retrospective | 25 | 35 | 4.5 |  |  | 3 |
| Alpert et al. 1994 [114] | Retrospective | 39 | 37 | 4.6 |  |  | 3 |
| Alpert et al. 1993 [20] | Retrospective | 39 | 37 | 4.6 |  |  | 3 |
| Alaud-din et al. 1990 [115] | Prospective | 12 | 37 | 13 | 50 |  | 4 |
